# TSC22D1 is a newly identified inhibitor of insulin secretion in pancreatic beta cells

**DOI:** 10.1111/febs.70194

**Published:** 2025-07-18

**Authors:** Sümbül Yıldırım, Amit Mhamane, Svenja Lösch, Annika Wieder, Ezgi Ermis, Ann‐Christine König, Sevgican Yilmaz, Stefanie M. Hauck, Fatih Kocabas, Julia Szendroedi, Stephan Herzig, Bilgen Ekim

**Affiliations:** ^1^ Department of Endocrinology and Metabolism, Joint Heidelberg IDC Translational Diabetes Program Heidelberg University Hospital Heidelberg Germany; ^2^ Department of Genetics and Bioengineering Faculty of Engineering, Yeditepe University Istanbul Türkiye; ^3^ Institute for Diabetes and Cancer (IDC), Helmholtz Diabetes Center, Helmholtz Center Munich Neuherberg Germany; ^4^ German Center for Diabetes Research (DZD) Neuherberg Germany; ^5^ Metabolomics and Proteomics Core (MPC), Helmholtz Center Munich Neuherberg Germany

**Keywords:** FoxO1, pancreatic beta cells and insulin secretion, TSC22D1

## Abstract

The loss of pancreatic beta cell function leads to chronically high blood glucose levels, contributing to diabetes mellitus, one of the leading causes of morbidity and mortality worldwide. Understanding the molecular mechanisms that regulate beta cell function could pave the way for the development of more effective antidiabetic treatments. In this study, we identify the evolutionarily conserved transforming growth factor β‐1 stimulated clone D1 (TSC22D1) protein as a previously unknown regulator of beta cell function. TSC22D1 depletion in INS‐1E cells enhances the expression of key beta cell identity genes, including *Ins1*, *Ins2*, *Pdx1*, *Slc2a2*, and *Nkx6.1*, and promotes glucose‐stimulated insulin secretion without altering intracellular insulin content. Mechanistically, TSC22D1 and Forkhead box protein O1 (FoxO1) interact and regulate each other in a reciprocal manner to control beta cell function. Our follow‐up interactome and RNA‐Seq analyses reveal that TSC22D1 is crucial for glucose‐responsive cellular processes in beta cells, including mRNA processing, ribonucleoprotein complex biogenesis, and Golgi vesicle transport. Overall, our findings indicate that TSC22D1 plays a significant role in regulating beta cell function at multiple levels, with potential implications for metabolic diseases, such as diabetes.

## Graphical abstract

TSC22D1 acts downstream of glucose signaling and negatively regulates insulin secretion in pancreatic beta cells. Mechanistically, TSC22D1 interacts with FoxO1. Elevated glucose levels enhance the TSC22D1–FoxO1 interaction indirectly by increasing the protein abundance of TSC22D1. Importantly, TSC22D1 and FoxO1 not only physically associate but also co‐regulate each other's function, controlling the expression of beta cell identity genes. Collectively, our findings identify TSC22D1 as a previously unrecognized regulator of beta cell function.

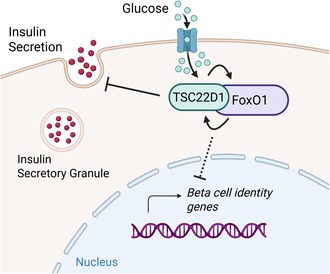

**Abbreviations**
co‐IP: co‐immunoprecipitation
Ddx1: DEAD‐box helicase 1
Ddx46: DEAD‐box helicase 46
DM: diabetes mellitus
EGF: epidermal growth factor
FBS: fetal bovine serum
FoxO1: Forkhead box protein O1
GSIS: glucose‐stimulated insulin secretion
HDL: high‐density lipoprotein
hnRNP A2/B1: heterogeneous nuclear ribonucleoprotein A2/B1
Hnrnpm: heterogeneous nuclear ribonucleoprotein M
IDF: International Diabetes Federation's
IF: immunofluorescence
Igfr1: insulin‐like growth factor receptor
IGFs: insulin‐like growth factors
Ins1: insulin 1
Ins2: insulin 2
Irs1: insulin receptor substrate 1
LC–MS/MS: liquid chromatography/tandem mass spectrometry
MafA: MAF BZIP transcription factor A
MAPK: mitogen‐activated protein kinase
mRNA: messenger RNA
mTORC1: mechanistic target of rapamycin complex 1
*n*
: sample size
NAFLD: nonalcoholic fatty liver disease
NeuroD1: neurogenic differentiation 1
Nkx6.1: homeobox protein
O/N: overnight
OE: overexpression
PARP: poly(ADP‐ribose)‐polymerase
PCA: principal component analysis
Pdx1: pancreatic and duodenal homeobox 1
Ptbp1: polypyrimidine tract binding protein 1
PTEN: phosphatase and tensin homolog
RNA‐Seq: RNA sequencing
RT: room temperature
Rtcb: RNA 2′,3′‐cyclic phosphate and 5′‐OH ligase
RT‐qPCR: quantitative reverse transcription polymerase chain reaction
Scgn: secretagogin
siRNA: scramble control
Slc2a2: solute carrier family 2 member 2
Slc2a4: solute carrier family 2 member 4
Tbp: TATA‐binding protein
TGFβ1: transforming growth factor‐beta 1
TSC22: transforming growth factor β‐1 stimulated clone D1
Ubqln1: ubiquilin 1
VCP: valosin‐containing protein
WCL: whole cell lysates

## Introduction

Diabetes mellitus (DM) is a chronic metabolic disorder characterized by abnormally high glucose levels due to impaired insulin action and/or production. According to the International Diabetes Federation's (IDF) Diabetes Atlas 2021, 537 million people suffered from diabetes in 2021, which is likely to reach 643 million by 2030 [1].

Pancreatic beta cells play an important role in glucose homeostasis by producing and secreting insulin to regulate blood glucose levels. In response to elevated blood glucose levels, beta cells not only upregulate insulin release but also suppress glucagon secretion from neighboring alpha cells, creating a coordinated hormonal response. A decline in insulin secretion and beta cell function has a critical role in the progression of type 2 DM [2]. Transcription factors, such as pancreatic and duodenal homeobox 1 (PDX1), neurogenic differentiation 1 (NeuroD1), MAF bZIP transcription factor A (MafA), and Homeobox protein Nkx6.1, regulate the expression of insulin and other genes involved in beta cell development, differentiation, and function [3]. The transcribed mRNA for insulin and other proteins involved in secretion is stored in RNA granules, also known as stress granules, with the help of RNA‐binding proteins, such as polypyrimidine tract binding protein (Ptbp1), DEAD‐box helicase 1 (Ddx1), and heterogeneous nuclear ribonucleoprotein (hnRNP) A2/B1. These stress granules provide mRNA stability and facilitate rapid translation, followed by the formation of insulin‐containing secretory granules, which are secreted upon glucose stimulation [4, 5, 6, 7, 8].

Members of the evolutionarily conserved transforming growth factor‐β1‐stimulated clone 22 (TSC22) protein family control several biological processes, such as cell growth and proliferation, differentiation, and apoptosis [9, 10, 11, 12, 13, 14, 15, 16, 17]. The TSC22 protein family is composed of four members: TSC22D1, TSC22D2, TSC22D3, and TSC22D4. They all share a domain structure known as the TSC domain, which includes a leucine zipper motif that enables them to form homodimers or heterodimers with one another [11, 18, 19]. Recently, our laboratory identified hepatic TSC22D4 as an environmental sensor that contributes to diabetic hyperglycemia, insulin resistance exacerbating nonalcoholic fatty liver disease (NAFLD) and liver fibrosis associated pathologies [20, 21, 22, 23]. Mechanistically, TSC22D4 interacts with Akt kinase, and elevated glucose levels impair TSC22D4–Akt interaction [20], which prompted us to investigate whether TSC22 proteins also regulate pancreatic beta cell function, where glucose sensing is key to regulate insulin secretion. The study we present here specifically focuses on TSC22D1 function in pancreatic beta cells.

TSC22D1 was the first TSC22 protein to be isolated and cloned from mouse osteoblast cells and identified as a TGF‐β induced gene [19]. In addition to TGF‐β, epidermal growth factor (EGF) and insulin‐like growth factors (IGFs) as well as glucocorticoids and cellular stress promote TSC22D1 expression to control differentiation, apoptosis, and proliferation in distinct cell types, such as hepatocytes, fibroblasts, neurons, and epithelial cells [9, 14, 24, 25]. Follow‐up studies identified TSC22D1 as a tumor suppressor gene that is downregulated in various cancers [26, 27, 28, 29]. Overexpression of TSC22D1, on the contrary, induces apoptosis [9, 13, 25]. Aside from its functions described in carcinogenesis, hepatic TSC22D1 also regulates high‐density lipoprotein (HDL) cholesterol levels via downregulation of transcriptional pathways that play a role in HDL formation [30]. TSC22D1 assumes a transcriptional co‐regulator role by interacting with several transcription factors to regulate gene expression. For example, it interacts with the transcription factor Smad4, a key mediator of the TGF‐β signaling pathway, to induce erythroid cell differentiation or interacts with c‐myc to inhibit its recruitment on the P15 and P21 promoters to regulate cell proliferation [10, 31]. The function of TSC22D1 in pancreatic beta cells, however, remains elusive.

Forkhead box O‐1 (FoxO1) acts as an important transcription factor as a regulator of critical cellular processes with implications in aging and diseases, such as metabolic disorders and cancer [32, 33, 34]. FoxO1 also plays an important role in regulating beta cell function for maintaining systemic glucose homeostasis [35, 36, 37]. FoxO1 directly binds to the promoters of *Pdx1* and *Insulin 2* (*Ins2*) genes to regulate beta cell function [38, 39]. In parallel, FoxO1 also regulates the expression of genes involved in insulin granule exocytosis to control insulin secretion in response to glucose stimulation [40]. Beta cell‐specific loss of FoxO1 induces dedifferentiation of beta cells into alpha cells, reducing beta cell mass, causing hyperglycemia and promoting hyperglucagonemia [36]. Indeed, triple knockout of Foxo genes (1, 3a and 4) causes a maturity‐onset diabetes of the young (MODY)‐like phenotype in mice [35]. Critically, FoxO1 function in beta cells depends on insulin signaling and nutrient availability, allowing beta cells to adapt to environmental conditions and needs [37]. While some studies suggest a protective role for FoxO1 in maintaining beta cell function, others, on the contrary, indicate that FoxO1 exacerbates the diabetic phenotype. Other transcription factors that control insulin gene expression include PDX11, NeuroD1/Beta2, Nkx6.1, MafA, hepatocyte nuclear factor (HNF) 1α, HNF 4α and Islet‐1 [3, 41, 42]. To ensure the precise regulation of insulin gene expression, these transcription factors interact within a complex regulatory network that is not yet fully understood.

Here, we present a comprehensive investigation of the molecular mechanisms underlying insulin secretion and beta cell function, with a specific focus on TSC22D1 protein. Based on our findings, TSC22D1 emerges as a novel key player acting as a negative regulator of insulin secretion from pancreatic beta cells. Our study provides compelling evidence that TSC22D1 regulates insulin secretion via the FoxO1 transcriptional network. Interestingly, TSC22D1 and FoxO1 interact and reciprocally regulate each other's function to control *Ins1* and *Ins2/Pdx1* gene expression, respectively. To shed further light on TSC22D1's mechanism of action, we additionally performed liquid chromatography/tandem mass spectrometry (LC‐MS/MS) to identify TSC22D1 partner proteins that show differential binding upon high glucose stimulation. These included several RNA‐binding proteins, such as Hnrnpm and Ddx46, as well as distinct tubulin isoforms, heat shock proteins, and Secretagogin (Scgn), a well characterized regulator of insulin secretion [43, 44, 45]. To further investigate the role of TSC22D1 in cellular response to glucose stimulation, we performed RNA‐sequencing (RNA‐Seq) experiments in the absence or presence of high glucose stimulation. The RNA‐Seq results strongly agreed with TSC22D1 interactome data and revealed ribonucleoprotein complex biogenesis, Golgi vesicle transport, and mRNA processing within the most deregulated pathways upon glucose stimulation in TSC22D1 deficient cells. Overall, by unraveling the intricate regulatory pathways governing insulin secretion and beta cell function, we aim not only to deepen our understanding of the pathogenesis of DM but also to provide critical insights for the development of novel therapeutic strategies targeting TSC22D1‐mediated pathways.

## Results

### TSC22D1 negatively regulates insulin secretion

**Fig. 1:**
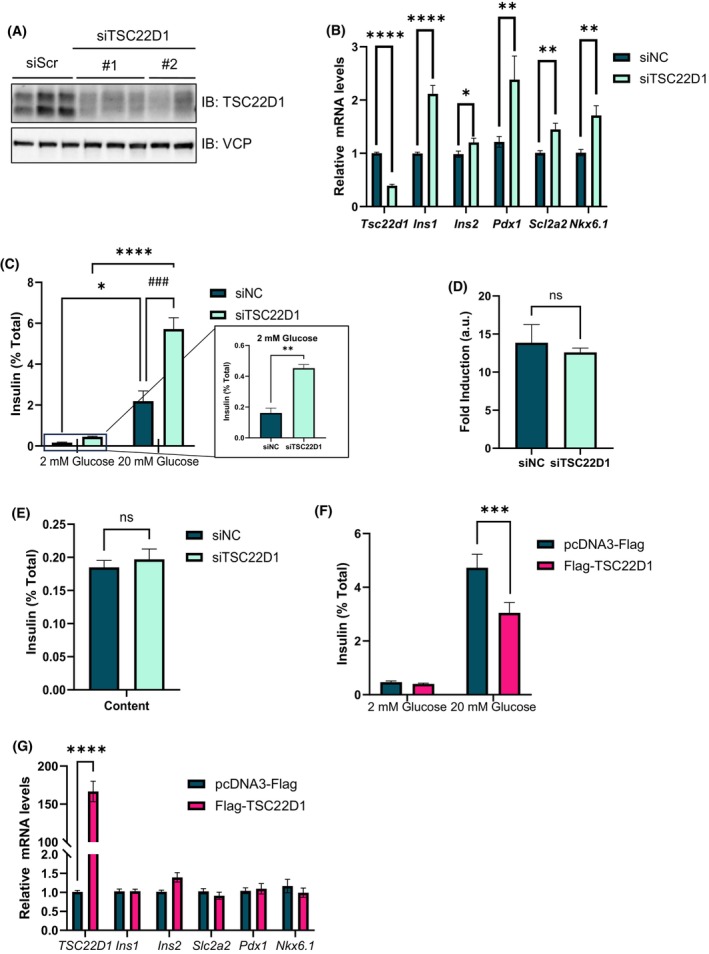
TSC22D1 regulates expression of beta cell identity genes and insulin secretion. (A) INS‐1E cells were transiently transfected with scrambled control [50 nm] vs. two different (#1 and #2) TSC22D1 siRNAs [50 nm]. Seventy‐two hours post‐transfection, cells were lysed and protein was extracted for western blotting with indicated antibodies. Data representative from two independent experiments each performed with three technical replicates. (B) INS‐1E cells were transiently transfected with scrambled control [50 nm] vs. TSC22D1 [50 nm] siRNAs. Seventy‐two hours post‐transfection, cells were lysed and RNA was extracted for reverse transcription polymerase chain reaction (RT‐PCR) analysis with indicated TaqMan probes. *n* = 13 for *TSC22D1* and *Ins1*, *n* = 10 for *Ins2*, *Pdx1*, *Slc2a2*, and *n* = 7 for *Nkx6.1* from four, three, and two independent experiments, respectively. (C) INS‐1E cells were transiently transfected with [50 nm] scrambled control vs. TSC22D1 siRNAs. Seventy‐two hours post‐transfection, cells were starved for 1 h in Tanaka Robertson Krebs Ring buffer (KRB) containing 0.1% bovine serum albumin (BSA) followed by stimulation with 2 mm and 20 mm glucose for 1 h. Medium and cells were collected for measuring secreted and intracellular insulin concentration using mouse insulin ELISA, respectively. Secreted insulin levels were normalized to total insulin content shown in (E). *Right panel*: Bar graph for insulin secretion at 2 mm glucose levels. (D) Fold induction of insulin secretion in response to glucose stimulation for data presented in (C). (E) Intracellular insulin content of (C) normalized to total protein. (C–E: *n* = 9; data representative of three independent experiments each performed with three technical replicates). (F) INS‐1E cells were transiently transfected with pcDNA3‐Flag vector control and Flag‐TSC22D1 plasmids. Forty‐eight hours post‐transfection, cells were starved for 1 h in Tanaka Robertson KRB with 0.1% BSA. Subsequently, cells were treated with 2 mm and 20 mm glucose for 1 h and medium was collected afterward. Mouse insulin ELISA was used for measuring insulin concentration. Secreted insulin was normalized to total insulin content. *n* = 18 from three independent experiments each performed with six technical replicates G. INS‐1E cells were transiently transfected with pcDNA3‐Flag vector control and Flag‐TSC22D1 plasmids. Forty‐eight post‐transfection, RNA was extracted for real‐time RT‐PCR analysis which was performed with indicated TaqMan probes. *n* = 18 for all targets from three independent experiments, each performed with six technical replicates (except for *Slc2a2; n* = 12 from two independent experiments). For the statistical analysis of the data presented in B, C, F, and G, two‐way ANOVA with Tukey's multiple comparisons tests was performed to determine the significance of differences observed between groups. For the inset in C, and for D and E, Student's *t*‐test was performed. *P < 0.05, **P < 0.01, ***P < 0.001, ****P < 0.0001. ^###^ *P* < 0.001 indicates the effect of TSC22D1 knockdown in [20 mm] glucose treated cells. Error bars represent SEM for all graphs.

Insulin synthesis and secretion by pancreatic β‐cells is a tightly regulated process both at the transcriptional and posttranslational level to maintain systemic glucose homeostasis. To test whether TSC22D1 regulates beta cell function, we knocked down TSC22D1 in INS‐1E cells and observed that TSC22D1 knockdown elevated the expression of insulin genes (*Ins1* and *Ins2*) as well as beta cell identity genes *Pdx1*, *solute carrier family 2 member 2* (*Slc2a2*, *i.e*., gene coding for GLUT2 protein) and *Nkx6.1* (Fig. 1A,B). Next, we performed a glucose‐stimulated insulin secretion (GSIS) assay to address whether TSC22D1 regulates insulin secretion. As shown in Fig. 1C, TSC22D1 deficient cells secreted more insulin compared with control cells in response to both low [2 mm] and high glucose [20 mm] treatments without any change in fold induction of insulin secretion and intracellular insulin content (Fig. 1C–E). As opposed to the loss‐of‐function approach with siRNA mediated‐TSC22D1 knockdown, we also overexpressed pcDNA3‐Flag vector control or Flag‐TSC22D1 in INS‐1E cells followed by GSIS assay. In contrast to TSC22D1 depletion, TSC22D1 overexpression suppressed GSIS compared with the vector control group without affecting the expression of *Ins1*, *Ins2*, *Pdx1*, *Slc2a2*, and *Nkx6.1* (Fig. 1F,G). Overall, our data indicate that TSC22D1 knockdown upregulates the expression of beta cell identity genes and promotes GSIS, which is impaired upon TSC22D1 overexpression.

### Transcriptomic profiling reveals differential regulation of signaling pathways upon TSC22D1 knockdown

For a further mechanistic insight on how TSC22D1 regulates beta cell function, we performed RNA‐sequencing (RNA‐Seq) analysis upon TSC22D1 knockdown under steady state conditions.

**Fig. 2:**
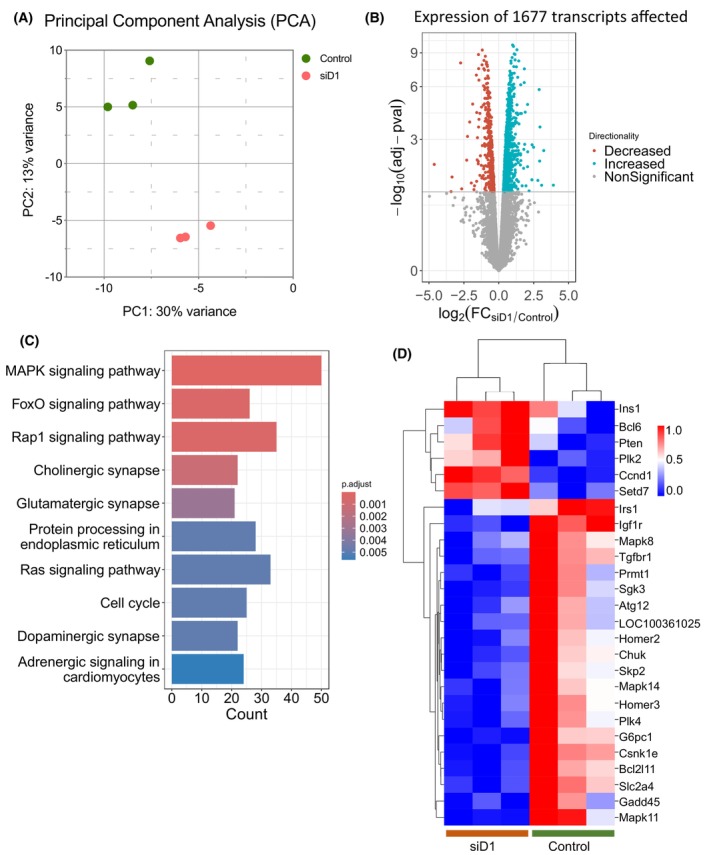
RNA‐Seq analysis in Control vs. TSC22D1 knockdown INS‐1E cells under steady state conditions. (A) Principal component analysis of RNA‐Seq data obtained from scrambled control [50 nm] and siTSC22D1 [50 nm] knockdown INS‐1E cells (siD) under steady‐state conditions. (B) Volcano plot of significantly differentially regulated transcripts in INS‐1E cells upon TSC22D1 knockdown. (C) KEGG Enrichment pathway analysis of differentially expressed genes. (D) Heatmap analysis of genes that are enriched within the FoxO1 signaling pathway. This experiment is performed in triplicates.

Our results showed that TSC22D1 knockdown deregulated the expression of 1677 transcripts under steady state conditions (Table S1, Fig. 2A,B) and the KEGG enrichment analysis revealed mitogen‐activated protein kinase (MAPK) and Ras signaling pathways, suggesting that TSC22D1 might be playing a role in controlling beta cell proliferation (Fig. 2C). Furthermore, the enrichment of the protein processing pathway in the endoplasmic reticulum indicates that perhaps TSC22D1 also plays a role in the regulation of insulin maturation and secretion at the posttranslational level (Fig. 2C). FoxO1, on the contrary, emerged as the only transcription factor potentially acting downstream of TSC22D1 (Fig. 2C). The deregulated targets that were allocated within the FoxO1 signaling pathway included Ins1, as well as regulators of the insulin signaling pathway, such as insulin receptor substrate (Irs1), phosphatase and tensin homolog (PTEN), and insulin‐like growth factor receptor 1 (Igfr1) as well as regulators of glucose metabolism, including Solute carrier family 2a4 (Slc2a4) and glucose‐6‐phosphatase (Fig. 2D). Overall, the RNA‐Seq analysis not only confirms our initial finding that TSC22D1 regulates *Ins1* gene expression (Fig. 1A) but also unravels FoxO1‐mediated gene regulation as one of the effectors of TSC22D1 function in the control of the insulin signaling pathway and glucose metabolism.

### TSC22D1 interacts with FoxO1

**Fig. 3:**
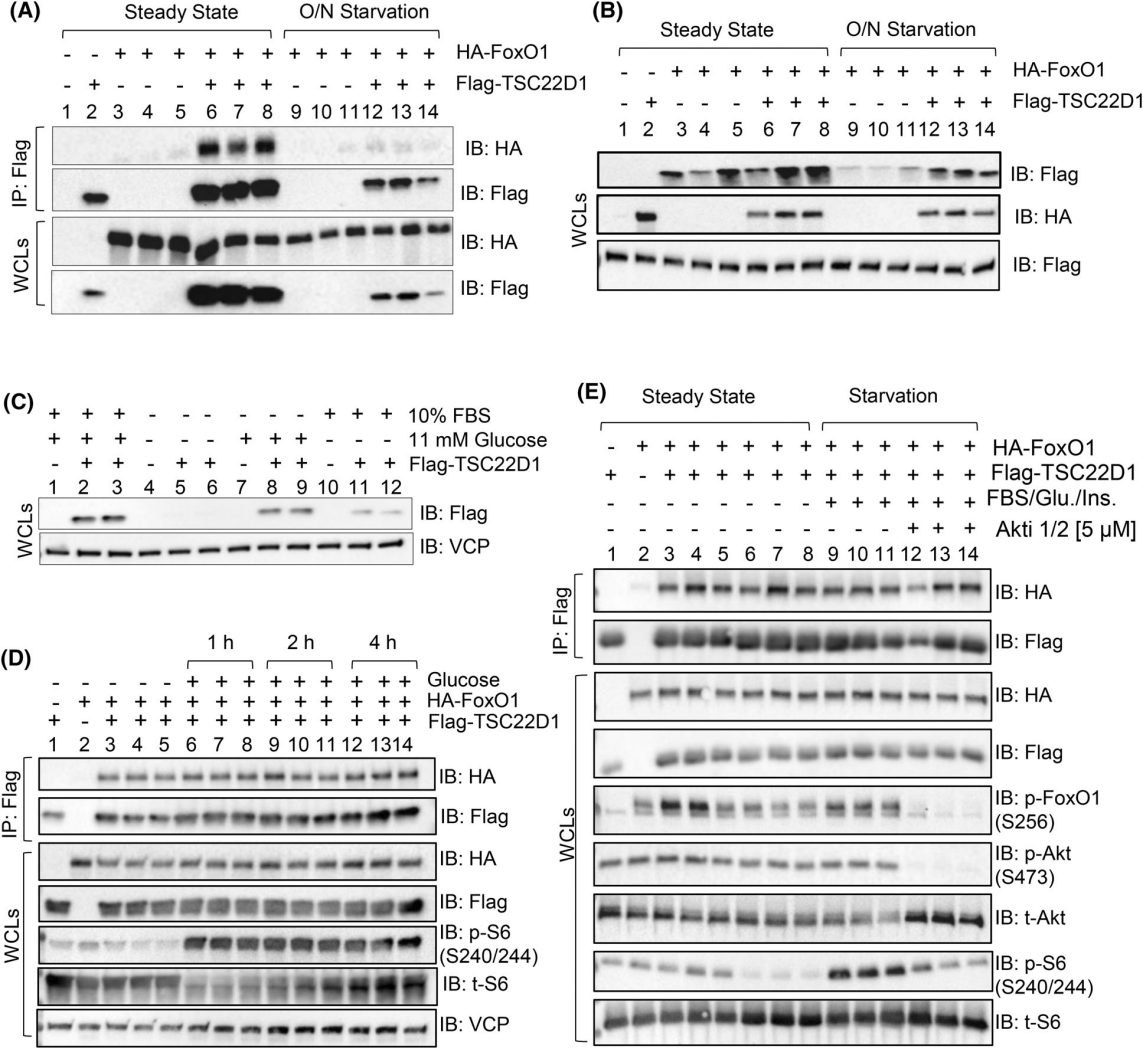
TSC22D1 interacts with FoxO1. (A) INS‐1E cells were transiently transfected with vector control (pcDNA3‐Flag) or co‐transfected with Flag‐TSC22D1 and HA‐FoxO1 plasmids. Thirty‐six post‐transfection, cells were either kept at steady state conditions or starved in the absence of fetal bovine serum (FBS) and glucose for 16 h (overnight, O/N). After cell lysis, Flag‐TSC22D1 was immunoprecipitated (IP) with anti‐Flag affinity gel, and the IPs and whole cell lysates (WCLs) were immunoblotted (IB) with indicated antibodies. (B) Similar to A without IPs. (C) INS‐1E cells were transiently transfected with vector control (pcDNA3‐Flag) or transfected with Flag‐TSC22D1 plasmids. Thirty‐six hours post‐transfection, cells were either kept at steady state conditions or starved in the absence of FBS and glucose together or individually for 16 h (overnight, O/N). WCLs were IB with indicated antibodies. (D) INS‐1E cells were transiently transfected with vector control (pcDNA3‐Flag) or co‐transfected with Flag‐TSC22D1 and HA‐FoxO1 plasmids. Forty‐eight hours post‐transfection, cells were starved in the absence of FBS and glucose for 4 h followed by glucose stimulations [20 nm] for 1, 2, and 4 h. After cell lysis, Flag‐TSC22D1 was IP with anti‐Flag affinity gel, and the IPs and WCLs were IB with indicated antibodies. (E) Similar to D except that cells were either kept under steady state conditions or starved in the absence of FBS and glucose for 4 h. Starved cells were pretreated in the presence or absence of Akti 1/2 [5 μm] for 1 h followed by FBS [10%], glucose [20 mm] and insulin [100 nm] stimulations for 1 h. After cell lysis, Flag‐TSC22D1 was IP with anti‐Flag affinity gel, and the IPs and WCLs were IB with indicated antibodies. All western blots represent data from at least three different independent experiments.

Since we observed changes in the expression of beta cell identity genes at the transcriptional level upon TSC22D1 knockdown (Fig. 1A), we decided to follow up our project by taking a closer look at FoxO1 transcription factor among other candidates in the RNA‐Seq pathway analysis (Fig. 2) and investigate its potential role as a downstream effector of TSC22D1 function. Based on previously published studies that show TSC22D1 acts as a transcriptional regulator and interacts with nuclear proteins [10, 31, 46, 47], we asked whether TSC22D1 interacts with FoxO1 transcription factor, too. To this end, we transiently transfected INS‐1E cells with Flag‐TSC22D1 and HA‐FoxO1 plasmids and performed co‐immunoprecipitation (co‐IP) experiments under two different conditions: steady state and overnight starvation, that is, in the absence of fetal bovine serum (FBS) and glucose. Western blot analysis of Flag pulldown confirmed the specific interaction between TSC22D1 and FoxO1 (Fig. 3A). Notably, overnight fasting impaired the TSC22D1–FoxO1 interaction, which was accompanied by a concomitant decrease in TSC22D1 protein levels in the whole cell lysates (WCL) (Fig. 3A, compare lanes 6–8 and 12–14). Overexpression of FoxO1, on the contrary, rescued starvation‐induced decrease in TSC22D1 protein levels (Fig. 3B, compare lanes 9–11, 12–14). In the follow‐up experiments, we addressed whether glucose or FBS deficiency in the starvation medium is responsible for reduced TSC22D1 protein levels. To this end, we starved the cells either in the absence of FBS or glucose alone or in the absence of both for 16 h. As shown in Fig. 3C, glucose deficiency alone reduced TSC22D1 protein levels to a greater extent compared with FBS depletion alone. Starving the cells in the absence of both FBS and glucose, however, caused the most substantial reduction on Flag‐TSC22D1 levels (Fig. 3C, compare Lanes 8–9 to 11–12), identifying nutrient availability as an upstream regulator of TSC22D1 protein levels.

Next, we asked whether glucose stimulation of starved cells regulates the TSC22D1–FoxO1 interaction. Since overnight FBS and glucose starvation reduces TSC22D1 protein levels (Fig. 3A), we starved the cells briefly for 4 h followed by glucose stimulation [20 nm] for 1, 2, and 4 h. Although glucose stimulation promoted mechanistic target of rapamycin complex 1 (mTORC1) signaling as we monitored by phosphorylation of its downstream target ribosomal protein S6 (S6), it had no effect on the TSC22D1–FoxO1 interaction (Fig. 3D). Similarly, starvation of the cells or stimulating the starved cells with FBS/insulin/glucose had no effect on the TSC22D1–FoxO1 interaction; while impairing or boosting S6 and FoxO1 phosphorylation, respectively (Fig. 3E, compare lanes 3–5 vs. 6–8 vs. 9–11). Additionally, blunting Akt kinase function by using the Akt inhibitor Akti 1/2 [5 μm], we again observed no effect on the TSC22D1–FoxO1 interaction (Fig. 3E).

Overall, these data indicate that TSC22D1 interacts with FoxO1 independent of FoxO1's phosphorylation status. Hence TSC22D1–FoxO1 interaction might represent a novel means of regulation of FoxO1 function and transcriptional network at least under steady state conditions as presented in Fig. 2. Furthermore, our results identify glucose and FBS availability as a regulator of TSC22D1 protein levels and suggest a protective role for FoxO1 to promote TSC22D1 protein stability, which might also be a function of TSC22D1–FoxO1 interaction.

### TSC22D1 and FoxO1 regulate each other in a reciprocal manner

**Fig. 4:**
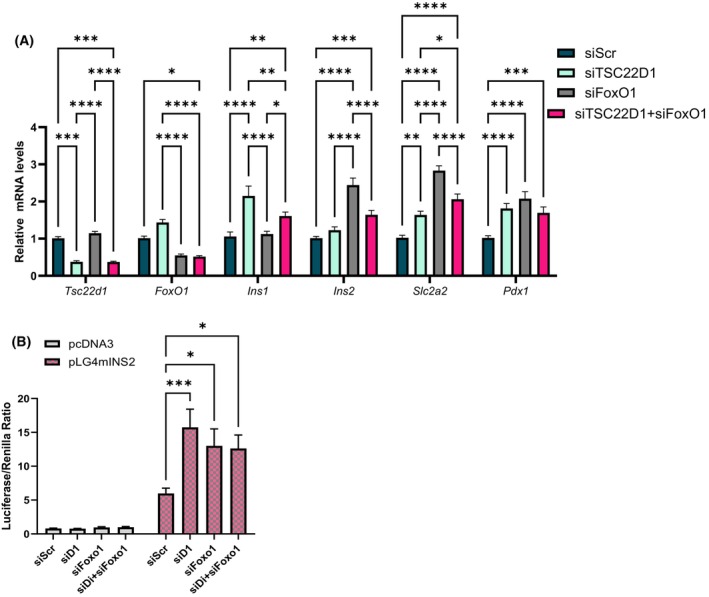
TSC22D1 and FoxO1 co‐regulate each other. (A) INS‐1E cells were transiently transfected with scrambled control siRNA (siScr) [50 nm] or siRNAs targeting TSC22D1 [50 nm], FoxO1 [50 nm] individually or TSC22D1 [50 nm] and FoxO1 [50 nm] together. Forty‐eight hours post transfection, RNA was extracted for reverse transcription polymerase chain reaction (RT‐PCR) analysis performed with indicated TaqMan probes. *n* = 12 for *TSC22D1*, *Ins1*, *Ins2*, *Slc2a2*; *n* = 11 for *Pdx1* and *n* = 9 for FoxO1 from four, four and three independent experiments, respectively, each performed with three technical replicates, respectively. (B) INS‐1E cells were transiently transfected with scrambled control siRNA (siScr) [50 nm] or siRNAs targeting TSC22D1 [50 nm], FoxO1 [50 nm] individually or TSC22D1 [50 nm], and FoxO1 [50 nm] together. Twenty‐four post‐transfection, cells were transfected again with pcDNA3 [50 ng] or pGL4mINS2 [50 ng] plasmids. Cells were incubated for a further 36 h, and then, luciferase assay was carried out using Dual‐Luciferase® Reporter Assay System following the manufacturer's instructions. *n* = 16 from four independent experiments each performed with four technical replicates. For the statistical analysis, one‐way ANOVA was used for each target gene in (A) and two‐way ANOVA was used in (B) to determine the significance of differences observed between groups, *P < 0.05, **P < 0.01, ***P < 0.001, ****P < 0.0001. Error bars represent SEM.

After identifying FoxO1 as a binding partner for TSC22D1, we asked whether TSC22D1 controls FoxO1 function or vice versa. To this end, we conducted single and double knockdown experiments in INS‐1E cells that involved silencing TSC22D1 and FoxO1 individually or simultaneously to distinguish their mutual effects on the expression of beta cell identity genes. As shown in Fig. 4A, TSC22D1 deficiency alone elevated *Ins1* gene expression, which was blunted by simultaneous knockdown of FoxO1. Single FoxO1 knockdown alone, on the contrary, induced both *Ins2* and *Slc2a2* gene expression, which was this time blunted by simultaneous knockdown of TSC22D1 (Fig. 4A). *Pdx1* expression, on the contrary, elevated to the same extent upon individual or simultaneous knockdown of TSC22D1 and FoxO1 (Fig. 4A). Overall, these data indicate that TSC22D1 and FoxO1 work together but employ distinct molecular mechanisms to regulate the expression of *Ins1* vs. *Ins2/Slc2a2* vs. *Pdx1*.

Next, we asked whether TSC22D1 regulates FoxO1 dependent transcriptional repression at the *Ins2* promoter as well [39]. To this end, we conducted a series of luciferase reporter assays by knocking down TSC22D1 and/or FoxO1 in INS‐1E cells followed by overexpression of control and luciferase pGL4‐Ins2 reporter vectors. Silencing of both TSC22D1 and FoxO1 individually resulted in a significant increase in luciferase activity driven by the *Ins2* promoter compared with control cells (Fig. 4B). Simultaneous knockdown of TSC22D1 and FoxO1, on the contrary, did not increase *Ins2* promoter activity any further, indicating that TSC22D1 and FoxO1 act in a linear manner within the same pathway to repress *Ins2* promoter activity (Fig. 4B).

Overall, our results confirm our RNA‐Seq analysis and provide further functional evidence that TSC22D1 and FoxO1 act in a reciprocal manner to regulate the expression of beta cell identity genes.

### Glucose stimulation regulates TSC22D1 interaction partners

Our RNA‐Seq analysis, co‐IP experiments, and beta cell functional assays identify FoxO1 both as a downstream effector and upstream regulator of TSC22D1 function. Yet, the single or double TSC22D1/FoxO1 knockdown experiments indicate that there must be other effectors of TSC22D1 apart from FoxO1, as TSC22D1/FoxO1 double knockdown only partially rescued the effects in regulation of *Ins1* gene expression and insulin secretion (Fig. 4).

**Fig. 5:**
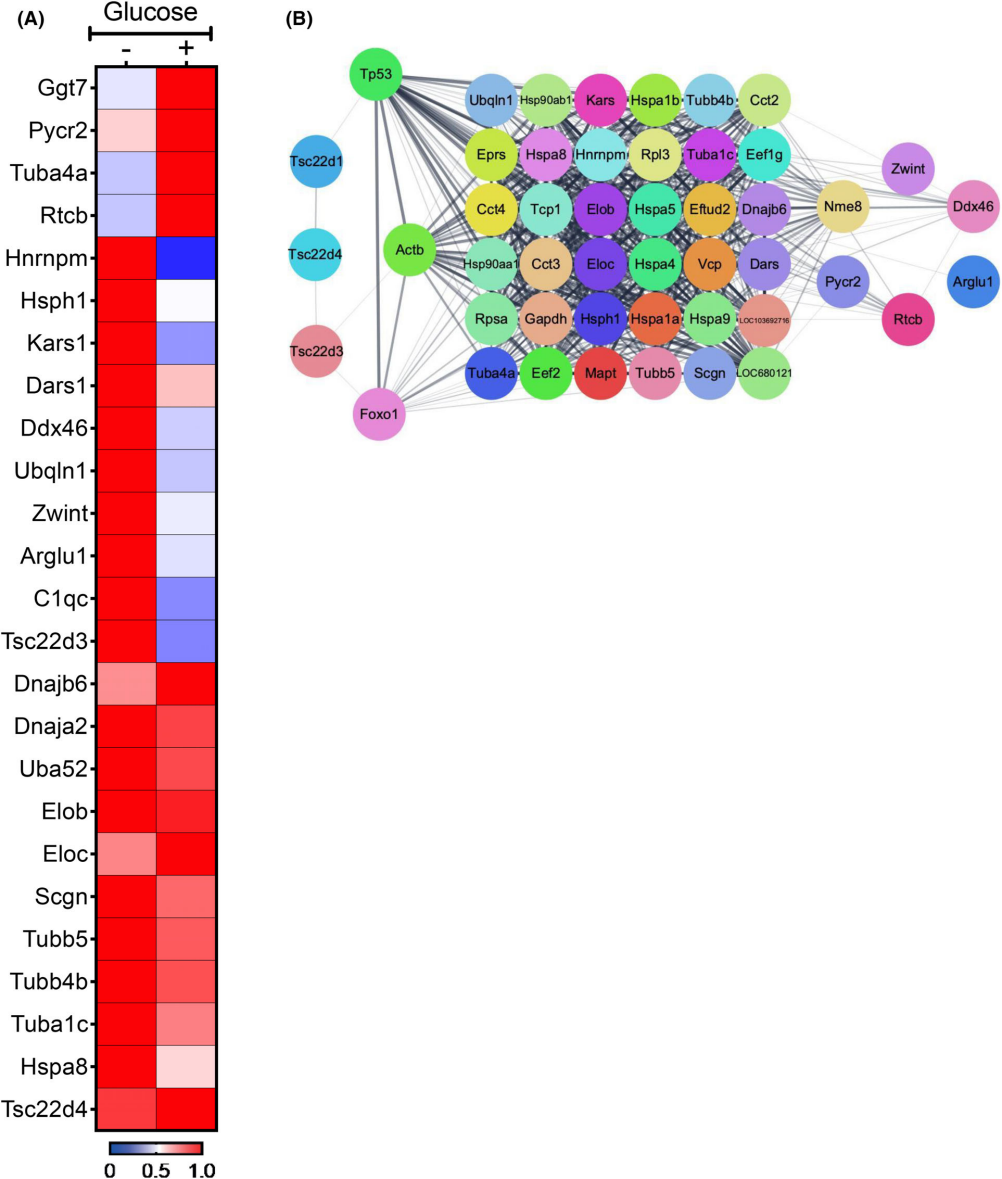
Identification of TSC22D1 binding partners via mass spectrometry. INS‐1E cells seeded on 10 cm dishes were transiently transfected with pcDNA3‐Flag vector control [10 μg] or Flag‐TSC22D1 [10 μg] plasmids. Forty‐eight hours post‐transfection, cells were briefly starved in Tanaka Robertson KRB for 1 h followed by 2 mm or 20 mm glucose stimulations for 1 h at 37 °C. Flag‐IP was performed with the cell lysates and IP eluates were subjected to LC‐MS/MS analysis. (A) Heatmap analysis of TSC22D1‐interacting proteins in the absence or presence of glucose stimulation. The enrichment of interacting proteins was normalized to vector control Flag‐IPs. The average of three replicates for each condition is represented. The scale bar refers to row peak normalized data in arbitrary units. (B) STRING Network [51] analysis of proteins identified in (A), including FoxO1 and known interaction partners.

Previous studies and now ours indicate that TSC22D1 regulates the function of its downstream targets via protein–protein interactions [10, 31, 46, 47]. To shed further light on the regulation of TSC22D1 function in beta cells, we aimed to identify TSC22D1's binding partners in INS‐1E cells and test whether they show differential binding upon glucose stimulation. To this end, we overexpressed Flag‐TSC22D1 in INS‐1E cells, which we starved briefly for 1 h followed by 1 h of [2 mm] or [20 mm] glucose stimulation. We lysed the cells and performed immunoprecipitation using anti‐Flag‐M2 affinity gel followed by LC‐MS/MS analysis to identify the binding partners. As shown in Fig. 5A, overall, we identified 23 different proteins that were specifically enriched in the Flag‐TSC22D1 IPs over the Flag‐only vector control. Ten out of twenty‐three proteins lost/weakened their interaction with TSC22D1 upon glucose stimulation, and 4 out of 23, on the contrary, showed more binding to TSC22D1 upon glucose stimulation. Glucose had no effect on TSC22D1's interaction with nine of the proteins, including TSC22D4, a well described interaction partner for TSC22D1 [11, 18], identifying them as rather constitutive binding partners (Fig. 5A). Furthermore, we identified both Elongin B (Elob) and Elongin C (Eloc) transcription elongation factors as constitutive partners of TSC22D1 (Fig. 5A). Interestingly, glucose stimulation abolished TSC22D1 interaction with RNA‐binding proteins heterogeneous nuclear ribonucleoprotein m (Hnrnpm), DEAD‐Box Helicase 46 (Ddx46), and RNA ligase Rtcb that control pre‐mRNA processing, mRNA metabolism, and transport [48, 49, 50]. Furthermore, our mass spectrometry analysis also identified Scgn as a TSC22D1 binding, protein, which has known functions in insulin secretion [23, 43, 44, 45]. The rest of the proteins that we identified also included tRNA synthetases (Dars1 and Kars1) as well as heat shock proteins Hsph1 and Hsp8a, suggesting that TSC22D1 indeed not only possesses nuclear functions but might also regulate cytoplasmic processes, such as translation and protein processing. The STRING Network analysis, including FoxO1 and other known interaction partners of the identified targets in Fig. 5A revealed that TSC22D3 and/or p53 might be mediating the TSC22D1–FoxO1 interaction (Fig. 5B) [51]. We also identified Eftud2, a nuclear protein playing role in pre‐mRNA processing and splicing as well as further ribosomal proteins, such as ribosomal protein L3 (Rpl3) and SA (Rpsa), together with eukaryotic translation elongation factor 1g (Eefg1) (Fig. 5B), providing further connection between TSC22D1 and not only in in (post)transcriptional but also in translational control (Fig. 5B). Other proteins that emerged in the STRING Network analysis included Microtubule associated protein (Mapt) (Fig. 5B), which suggest that together with tubulin alpha‐4A chain (Tuba4a), tubulin beta 5 class I (Tubb5), tubulin beta 4B class IVb (Tubb4b), and tubulin alpha 1C (Tuba1c) that we identified as TSC22D1 binding partners, TSC22D1 might regulate the transport of insulin secretory granules to the cell membrane [52, 53] (Fig. 5A,B).

### TSC22D1 predominantly localizes to the cytoplasm

**Fig. 6:**
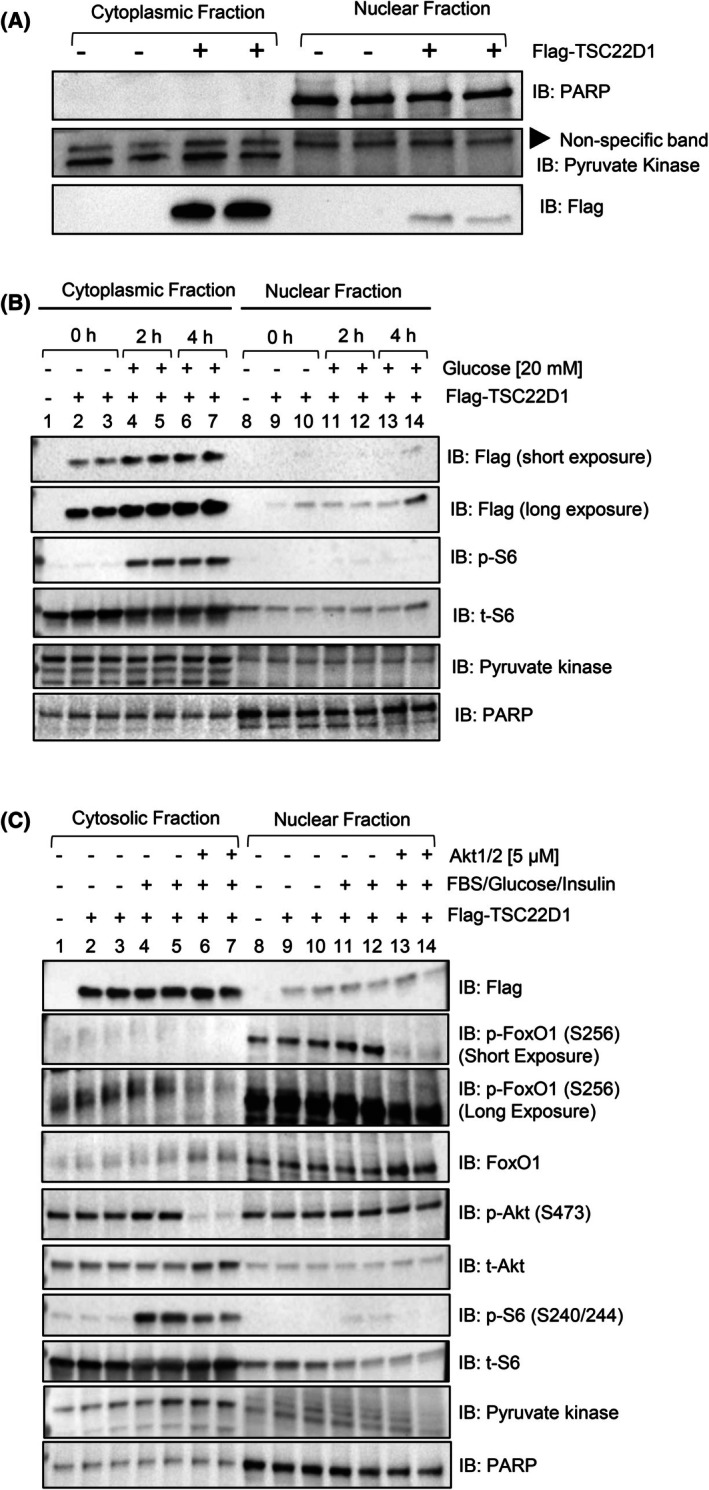
TSC22D1 predominantly localizes to the cytoplasm. (A) INS‐1E cells were transiently transfected with pcDNA3‐Flag vector control and Flag‐TSC22D1 plasmids. Forty‐eight hours post‐transfection, cells were lysed and a subcellular fractionation assay was performed. The protein concentration of cytosolic and nuclear fractions was normalized and analyzed by western blotting with indicated antibodies. (B) As in A, except that cells were starved in the absence of fetal bovine serum (FBS) and glucose for 4 h followed by glucose stimulations [20 nm] for 2 h and 4 h followed by cell lysis and subcellular fractionation. (C) Similar to B, except that FBS‐ and glucose‐starved cells were pretreated in the presence or absence of Akt inhibitor (Akti) 1/2 [5 μm] for 1 h followed by FBS [10%], glucose [20 mm] and insulin [100 nm] stimulations for 1 h. All western blots represent data from at least three different independent experiments.

Our mass spec analysis surprisingly revealed mostly cytoplasmic proteins as binding partners of TSC22D1 (Fig. 5). Hence, we investigated the subcellular localization of TSC22D1 initially under steady state conditions and observed that TSC22D1 predominantly localizes to the cytoplasm (Fig. 6A). Stimulation of the starved cells with glucose for 2 h and 4 h elevated the Flag‐TSC22D1 levels in the cytoplasmic fraction without an effect on nuclear fraction (Fig. 6B), indicating that the increase in cytoplasmic fraction is not necessarily due to a shuttling from nucleus to the cytoplasm but rather due to elevated TSC22D1 stability or impaired degradation, further supporting our data in Fig. 3C where glucose acts as a positive regulator of TSC22D1 protein levels (Fig. 6B). Additionally, stimulating the starved cells with FBS/insulin/glucose for 1 h did not affect TSC22D1's subcellular localization neither in the absence nor presence of Akti 1/2; where nuclear accumulation of FoxO1 in the nuclear compartment was evident in the latter (Fig. 6C; compare lanes 11–12 to 13–14).

### TSC22D1 deficient cells show altered glucose response in transcriptional network

**Fig. 7:**
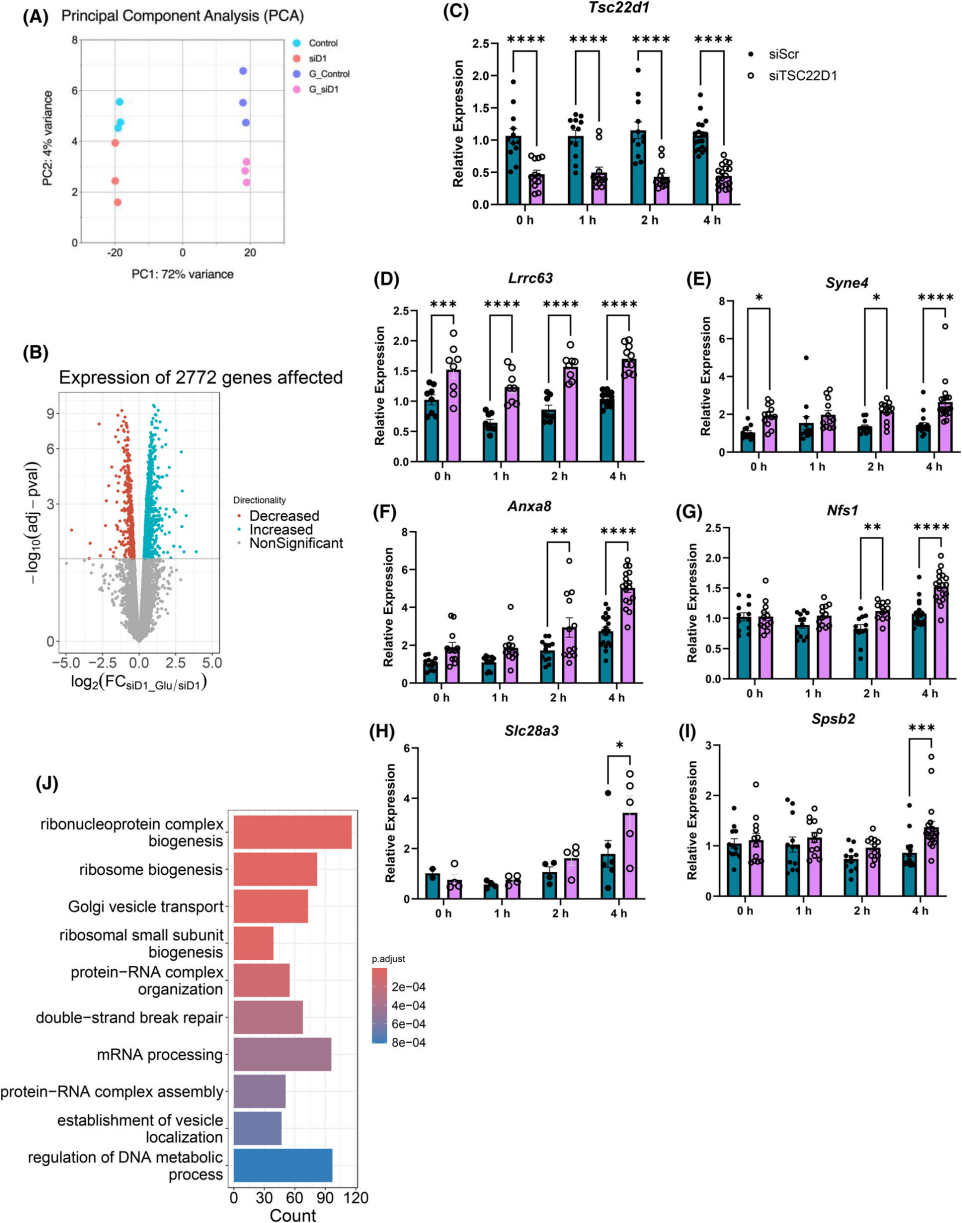
RNA‐Seq analysis in control vs. TSC22D1 knockdown INS‐1E cells upon glucose stimulation. (A) Principal component analysis of RNA‐Seq data obtained from scrambled control and TSC22D1 knockdown INS‐1E cells (siD) in the absence or presence of glucose stimulation [20 mm] (G_control, G_siD1) for 4 h. (B) Volcano plot of genes that are differentially regulated in TSC22D1 deficient but not in control INS‐1E cells upon glucose stimulation [20 mm] for 4 h. (A, B: *n* = 3 for each experimental group.) (C–I) INS‐1E cells were transiently transfected with [50 nm] scrambled control (dark gray bars) or TSC22D1 siRNAs (light gray bars). Seventy‐two hours post‐transfection, cells were starved in the absence of fetal bovine serum (FBS) and glucose for 4 h followed by glucose stimulation [20 nm] for 1, 2, and 4 h followed by cell lysis for RNA extraction and reverse transcription polymerase chain reaction (RT‐PCR) with indicated Taqman probes. *n* = 12 for all targets from three independent experiments each performed with four technical replicates except for Lrrc3 (*n* = 8) and Slc28a3 (*n* = 4 or 6) from two independent experiments. Two‐way ANOVA was used in C–I to determine the significance of differences observed between groups, *P < 0.05, **P < 0.01, ***P < 0.001, ****P < 0.0001. (J) GO enrichment pathway analysis of differentially expressed genes presented in (B). Error bars represent SEM.

The differential regulation of TSC22D1 interaction with their binding partners in the absence or presence of glucose suggests that TSC22D1 might act as a glucose sensor to control beta cell function. To this end, we repeated the RNA‐sequencing experiment in scrambled control vs. TSC22D1 knockdown cells upon glucose stimulation to address how TSC22D1 deficiency affects cellular response to high glucose treatment. The principal component analysis (PCA) demonstrated a robust effect of glucose stimulation on transcriptomic profile where control vs. TSC22D1 knockdown cells clustered differently (Fig. 7A). We identified 900 genes that were differentially down‐ or upregulated upon glucose stimulation in control‐ but not in TSC22D1‐knockdown cells (Table S2). Upon TSC22D1 knockdown, on the contrary, 2772 genes were differentially regulated with glucose stimulation which did not change at all in control cells (Fig. 7B; Table S3). To validate our RNA‐Seq data as well as to address whether the effects of TSC22D1 knockdown on target genes are indirect or direct, we performed qPCR analysis for selected target genes in control vs. TSC22D1 knockdown cells stimulated with glucose [20 nm] in a time course manner, that is, for 1, 2, and 4 h. Our results showed that some targets, such as leucine rich repeat containing 63 (*Lrrc63*) and spectrin repeat containing nuclear envelope family member 4 (*Syne4*), were already upregulated in TSC22D1‐deficient cells, independent of glucose stimulation (Fig. 7C–E). However, this may still be due to indirect effects, as TSC22D1 knockdown had already persisted for 72 h. For Annexin A8 (*Anxa8*) and NFS1 Cysteine Desulfurase (*Nfs1*) a statistically significant difference was observed as early as 2 h of glucose stimulation, whereas for solute carrier family 28 member 3 (*Slc28a3*) and SPRY domain‐containing SOCS box protein 2 (*Spsb2*), differences between control and TSC22D1 knockdown cells only became evident after 4 h (Fig. 7C–I). The pathway analysis of these differentially regulated genes that appeared only in TSC22D1 knockdown analysis upon glucose stimulation (Table S3) revealed again posttranscriptional processes, such as mRNA splicing, as well as formation of ribonucleoprotein complexes together with ribosomal regulation, indicating not only translational control but perhaps also stress granules. Further pathways also pointed toward Golgi vesicle transport and vesicle localization, which are critical functions for maturation of insulin secretory vesicles and their secretion (Fig. 7J); all of which align very well with our mass spectrometry analysis where we identify binding partners for TSC22D1 acting in all these cellular processes (Fig. 5).

**Fig. 8:**
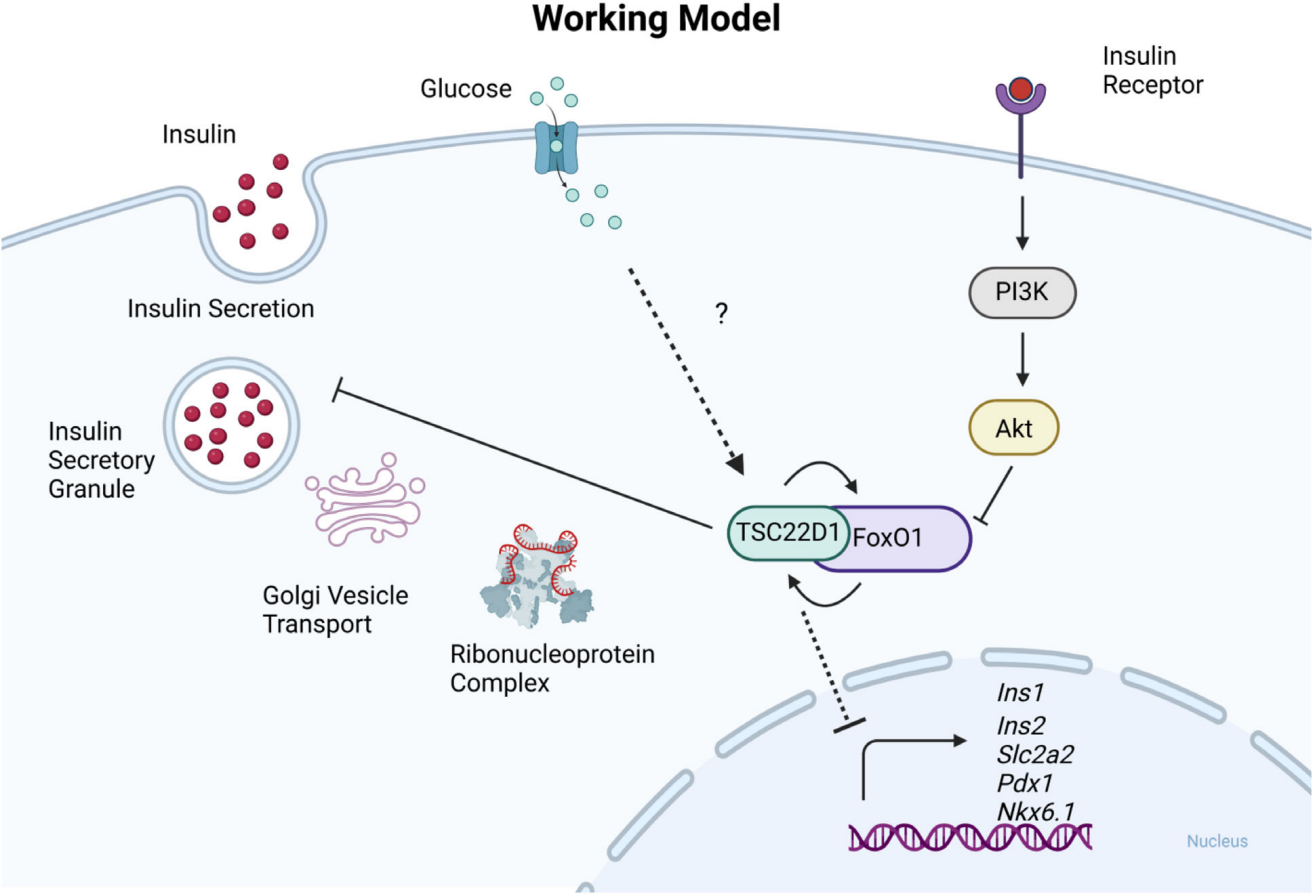
Working model. TSC22D1 and FoxO1 co‐regulate each other to control the transcription of beta cell identity genes. Given its predominant cytoplasmic localization, TSC22D1 likely plays an indirect role in transcriptional regulation. Glucose maintains TSC22D1 protein levels through unknown mechanisms. Additionally, TSC22D1 interacts with proteins involved in ribonucleoprotein complex assembly and Golgi vesicle transport, potentially contributing to its inhibitory effect on insulin secretion.

Overall, our findings indicate that TSC22D1 controls beta cell function at least at two levels, which we summarized in our working model (Fig. 8):

1. TSC22D1 and FoxO1 interact with each other and co‐regulate the expression of beta cell identity genes. The fact that TSC22D1 predominantly localizes to the cytoplasm suggests that the role of TSC22D1 in transcriptional control of beta cell identity genes takes place most likely in an indirect way.
2. By interacting with and controlling components of ribonucleoprotein complexes and vesicle transport, TSC22D1 might also control insulin secretion by regulating the formation and transport of secretory granules.

## Discussion

Here, we identify TSC22D1 as a novel regulator of beta cell function. Specifically, TSC22D1 acts as a negative regulator of beta cell identity gene expression and insulin secretion. To investigate its role in pancreatic beta cells, we knocked down TSC22D1 in INS‐1E cells and performed functional assays. Western blot analysis confirmed the knockdown at the protein level, revealing two TSC22D1 bands around 15 kDa, corresponding to two distinct transcripts with different ATG start codons. [54]. Interestingly, while TSC22D1 knockdown elevated the expression of *Ins1*, *Ins2* and beta cell identity genes, the intracellular insulin content did not increase, suggesting that there is a rate limiting step either at the translational step during the synthesis of preproinsulin or at the posttranslational processing step from preproinsulin to mature insulin (Fig. 1A–E). Nevertheless, TSC22D1 knockdown did elevate GSIS (Fig. 1C), suggesting that, in addition to impairing the expression of beta cell identity genes, TSC22D1 also plays an inhibitory role in insulin secretion itself, which was also evident in impaired insulin secretion upon TSC22D1 overexpression without an effect on expression of beta cell identity genes (Fig. 1F,G). This is further supported by our interactome analysis, where we identified Scgn and tubulin proteins, which aid in secretory granule transport, as TSC22D1 binding partners [45, 52, 55, 56, 57] (Fig. 5A). Additionally, Golgi vesicle transport and vesicle localization also emerged as enriched pathways in TSC22D1 knockdown cells upon glucose stimulation which might mediate TSC22D1's role in insulin secretion (Fig. 7J).

TSC22D1 acts through FoxO1—at least in part—to control the expression of *Ins1* (Fig. 4A). Furthermore, TSC22D1 knockdown elevates FoxO1 mRNA expression, suggesting the presence of compensatory mechanisms between the two proteins (Fig. 4A). Interestingly, TSC22D1 knockdown promotes *Ins1* gene expression at a much higher level compared with *Ins2* (Figs 1B and 4A), validating the presence of distinct transcriptional regulators that control *Ins1* vs. *Ins2* gene expression [39, 58]. (Our findings also point out that the functional connection between TSC22D1 and FoxO1 is reciprocal, i.e., FoxO1 depends on TSC22D1 too to suppress *Ins2* and *Slc2a2* gene expression; Fig. 4A). Interestingly, the FoxO1‐TSC22D1 interaction occurs not only at the functional but also at the physical level (Fig. 3), aligning well with previous studies that indicate a regulatory role in transcriptional control for TSC22D1 through interacting with other transcription factors [10, 18, 31, 46, 47]. Since TSC22D1 localizes mainly in the cytoplasm (Fig. 6), we speculate that the TSC22D1–FoxO1 interaction takes place most likely in the cytoplasm and the effects of TSC22D1 on transcription of beta cell identity genes must be rather indirect. This is further supported by evidence that, unlike knockdown, TSC22D1 overexpression did not cause changes in the expression of beta cell identity genes, indicating that TSC22D1 overexpression alone is not sufficient to suppress the expression of *Ins1*, *Ins2*, *Pdx1*, *Slc2a2*, and *Nkx6.1* genes, which might be due to rate limiting steps downstream of TSC22D1 action (Fig. 1G). Overall, we identify a reciprocal connection between TSC22D1 and FoxO1 that occurs at multiple levels to regulate beta cell‐specific functions. Interestingly, FoxO1 did not appear in our interactome analysis, suggesting that the TSC22D1–FoxO1 interaction might be indeed indirect and mediated by p53 or TSC22D3 (Fig. 5B). The TSC22D1–FoxO1 interaction might take place at a rather low stoichiometry, requiring FoxO1's overexpression for detection as well (Fig. 3A,D,E).

Furthermore, the lack of regulation of the TSC22D1–FoxO1 interaction by insulin, FBS, glucose stimulation, or Akt inhibition may be due to the fact that we detected this interaction under conditions of overexpression of both proteins (Fig. 3A,D,E). Despite multiple attempts, we were unable to successfully immunoprecipitate endogenous TSC22D1 using commercially available antibodies, limiting our ability to study the interaction at more physiological levels. Additionally, while Akt inhibition was successful, stimulation of the INS‐1E cells with FBS, insulin, and glucose resulted in only minimal increases in Akt and FoxO1 phosphorylation (Figs 3E and 6C). This outcome is not unexpected, as pancreatic beta cells have evolved mechanisms to desensitize insulin receptor signaling to protect against autocrine insulin effects, preventing uncontrolled cell growth and proliferation. One such mechanism involves the insulin inhibitory receptor (inceptor), which suppresses insulin‐induced Akt phosphorylation in wild‐type pancreatic beta cells. Notably, this phosphorylation becomes evident in inceptor KO beta cells, as reported recently [59]. Nevertheless, we could monitor the effects of FBS, insulin and glucose on S6 phosphorylation, which was still partially dependent on Akt activity (Figs 3E and 6C).

The immediate effectors of TSC22D1 upon glucose stimulation might also include its binding partners that show differential binding depending on the absence or presence of glucose, such as Ubiquilin 1 (Ubqln1) and RNA‐binding proteins Hnrnpm, Ddx46, and Rtcb (Fig. 5A). Both Hnrnpm and Ddx46 emerge as interesting candidates as Hnrnp A1/B2 and Ddx1 within the same protein family, respectively, interact with insulin mRNA and localize to the stress granules similar to Rtcb and Ubqln1, which we identified in our interactome analysis (Fig. 5A). The stress granules that contain these RNA‐binding proteins and mRNAs are resolved upon glucose stimulation to promote insulin synthesis and secretion [7, 8]. Hence, it is also prompting to speculate that perhaps by interacting with TSC22D4, which contains a long stretch of intrinsically disordered region that is found in RNA‐binding proteins [60], TSC22D1 also localizes to stress granules to form large ribonucleoprotein complexes to control the stability of mRNAs that play a role in insulin secretion. Indeed, the ribonucleoprotein complex biogenesis emerged as the most enriched deregulated pathway in our RNA‐Seq analysis in response to glucose stimulation in TSC22D1 knockdown cells (Fig. 7J). To amplify the effects of glucose stimulation in our transcriptomic and co‐IP analyses, we preincubated the cells in glucose‐free medium prior to glucose treatment. While this condition does not accurately recapitulate *in vivo* physiology and may impose cellular stress with prolonged exposure, we mitigated these potential effects by restricting the starvation period to 4 h, unless otherwise indicated.

Finally, starvation of the cells, particularly in the absence of glucose, downregulated TSC22D1 protein levels, which was rescued by overexpression of HA‐FoxO1 (Fig. 3A–C). Since we used a Flag‐TSC22D1 expression plasmid without any 5′ or 3′ untranslated regions (UTR), we conclude that HA‐FoxO1 must be controlling TSC22D1 protein levels not at the transcriptional or translational, but rather at the posttranslational level—most likely via increasing TSC22D1 stability or preventing its degradation (Fig. 3B). In support of this, we also observed a slight increase in Flag‐TSC22D1 protein levels upon 2 h and 4 h of glucose stimulation, which did not take place at the mRNA level (Figs 6B and 7C). Hence, we identify glucose as a positive regulator of TSC22D1 protein levels, the exact mechanism of which remains elusive (Fig. 8). This observation, however, remains paradoxical as one can ask why would glucose promote TSC22D1 protein levels if TSC22D1 has an inhibitory effect on insulin secretion? We can answer this question by suggesting that perhaps TSC22D1 simply acts within a feedback loop to fine‐tune insulin secretion, or it might also act as an inhibitor of insulin secretion upon prolonged hyperglycemia to prevent beta cell exhaustion. Nevertheless, targeting TSC22D1 in diabetic pancreatic beta cells might help recover beta cell function by promoting the expression of beta cell identity genes and insulin secretion.

In summary, we have identified TSC22D1 as a novel regulator of beta cell function that acts at multiple levels to suppress insulin synthesis and secretion, involving FoxO1 and other interaction partners identified through mass spectrometry. Our study provides critical insights into the role of TSC22D1 in beta cell function, highlighting its potential as a therapeutic target to enhance insulin secretion in diabetes. By deepening our understanding of the molecular pathways governing insulin biosynthesis, we are paving the way for new interventions in the treatment of both type 1 and type 2 diabetes and their long‐term complications.

The limitation of the study: Here, we employed *in vitro* systems to elucidate the molecular mechanisms by which TSC22D1 controls beta cell function. Additional research utilizing primary islets and beta cell‐specific TSC22D1 knockout mice is required to validate these findings *in vivo* and investigate the potential therapeutic benefits of TSC22D1 regulation. Furthermore, due to lack of commercially available antibodies suitable for TSC22D1 IP, we also overexpressed both Flag‐TSC22D1 and HA‐FoxO1 to detect their interaction, limiting our ability to study the interaction at more physiological levels.

## Materials and methods

### Cell culture

INS‐1E cells were a kind gift from Dr. Maria Rohm at the Institute for Diabetes and Cancer at Helmholtz Diabetes Center, Munich, Germany. INS‐1E rat cells were cultured in Roswell Park Memorial Institute 1640 Medium (RPMI 1640 Medium) + GlutaMAX™ (Gibco, Grand Island, NY, USA; #61870036), which contained a nutrient mixture of 10% FBS, 1% PSA (Penicillin–Streptomycin‐Amphotericin B Solution), 1% HEPES (1 m; Gibco, #15630080), and 1% Sodium Pyruvate (100 mm; Gibco, #11360070). INS‐1E cells were grown in an incubator with 5% CO_2_ that was humidified to 37 °C. All experiments were performed with mycoplasma‐free cells.

### RNA isolation, cDNA and RT‐PCR

Following the manufacturer's instructions, total RNA was isolated from INS‐1E cells using the QIAzol and RNeasy kit (QIAGEN, Hilden, North Rhine‐Westphalia, Germany; #74106). In order to synthesize cDNA, First Strand cDNA Synthesis Kit (Thermo Fisher Scientific, Waltham, MA, USA, #K1612) was used with 1 μg of RNA according to the manufacturer's instructions. Quantitative reverse transcription polymerase chain reaction (RT‐qPCR) was carried out with an ABI StepOnePlus sequence detector (Applied Biosystems) using the TaqMan gene expression assay (Thermo Fisher Scientific, #437048). Data were analyzed by using ΔΔCt method, and *TATA‐binding protein* (*Tbp*) was used as a housekeeping gene to normalize the results. The Assay IDs for each target are listed in Table 1.

**Table 1:** List of genes for qPCR analysis and their corresponding TaqMan Assay IDs (Thermo Fisher Scientific).

Gene name	Assay ID
*Tbp*	Rn01455646_m1
*Tsc22d1*	Rn00564852_m1
*Ins1*	Rn02121433_g1
*Ins2*	Rn01774648_g1
*Slc2a2*	Rn00563565_m1
*Pdx1*	Rn00755591_m1
*Nkx6.1*	Rn01450076_m1
*FoxO1*	Rn01494868_m1
*Anxa8*	Rn01756160_m1
*Slc28a3*	Rn00590238_m1
*Nfs1*	Rn02586518_m1
*Spsb2*	Rn02347348_g1
*Syne4*	Rn02346596_m1
*Lrrc63*	Rn02396724_m1

### Glucose‐stimulated insulin secretion assay

INS‐1E cells were seeded to 12‐well plates (300.000 cell/well) and after 24 h cells were transfected with 50 nm siRNA scramble control (QIAGEN, #1027281), siTSC22D1 (Thermo Fisher Scientific, #RSS363732 or Sigma‐Aldrich, St. Louis, MO, USA; #SASI_Rn02_00260758), and siFoxO1 (Thermo Fisher Scientific, #RSS331473) using Lipofectamine RNAiMAX (Invitrogen, Carlsbad, CA, USA; #13778150) and OPTIMEM (Gibco, #31985047). Seventy‐two hours post‐transfection, cells were washed twice with 1 mL/well Tanaka Robertson Krebs Ring Buffer (KRB) (118.5 mm NaCI, 2.54 mm CaCI_2_; 1.19 mm KH_2_PO_4_, 4.74 KCI, 25 mm NaHCO3; 1.19 mm MgSO_4_ 7H_2_O and 10 mm HEPES) containing 0.1% bovine serum albumin (BSA) with and starved in the presence of Tanaka Robertson KRB with 0.1% BSA for 1 h at 37 °C. Following starvation, the cells were treated with Tanaka KRB 0.1% BSA containing 2 mm or 20 mm glucose for 1 h at 37 °C. After glucose stimulation, cell medium was transferred to fresh Eppendorf tubes and centrifuged at 5000 g, 4 °C for 2 min. After centrifugation, 250 μL supernatant was transferred into new tubes and stored at −20 °C. For insulin content, remaining cells in the petri dishes were harvested by adding 250 μL 1.5% HCl in a 70% ethanol and stored at −80 °C for at least 2 h. Mouse Insulin ELISA kit (ALPCO, #80‐INSMS‐E10) was used for measuring secreted insulin and intracellular insulin content by following the manufacturer's instructions. Protein concentration was measured with the BCA Protein Assay Kit by following the manufacturer's instructions (Thermo Fisher Scientific, #23225). Each analysis was rigorously performed with at least three biological replicates to ensure that results accounted for biological variability. Within each biological replicate, at least three technical replicates were included to increase the precision and reliability of the measurements.

### Transient transfection and Co‐immunoprecipitation (co‐IP)

INS‐1E cells were co‐transfected with pENTR‐TSC22D1‐Flag (species mouse) [30], pCMV5‐HA‐FoxO1 (kind gift from Joan Massague; Addgene #14936; species mouse) [61] and pcDNA3‐Flag empty control plasmids using Lipofectamine™ 2000 Transfection Reagent (Invitrogen, #11668019) and OPTIMEM (Gibco, #31985047). Cells were lysed with ice‐cold BLB‐chaps lysis buffer 10 mm KPO4 (pH: 7.2), 10 mm EDTA, 5 mm EGTA; 10 mm MgCI_2_; 50 mm β‐Glycerophosphate, and 0.3% CHAPS containing protease (Roche, Penzberg, Bavaria, Germany; #11873580001) and phosphatase inhibitors (Thermo Fisher Scientific, #A32957) and centrifuged at 18 213 ***g***, 4 °C, for 5 min. After centrifugation, supernatants were collected, and protein concentration was measured with Bradford Assay (Bio‐Rad, Hercules, CA, USA; #5000006). Three hundred micrograms of protein was sufficient for co‐IP. For Co‐IP, cell lysates were incubated with anti‐FLAG M2 gel (Sigma‐Aldrich, #A2220‐5ML) for 2 h at 4 °C on a rotator. After incubation, the beads were washed three times with 1 mL ice‐cold lysis buffer, and finally, the gel beads were resuspended in 2× Laemmli sample buffer and boiled at 95 °C for 5 min. The co‐immunoprecipitation was subsequently analyzed by western blotting.

### Western blot analysis

Protein extracts and immunoprecipitates (IPs) were subjected to SDS/PAGE using any kD precast protein gels (Bio‐Rad, #4569036) and subsequently transferred to nitrocellulose membranes (Bio‐Rad, #2895). Membranes were blocked for 1 h at room temperature (RT) in 5% nonfat dried milk diluted in tris‐buffered saline (TBS) containing 1% Tween 20 (TBS‐T). Following the blocking step, membranes were incubated overnight at 4 °C on a rocking platform with primary antibodies specific for Flag‐tag (Sigma #A8592), HA‐tag (CST, #2999S), Poly(ADP‐ribose)‐Polymerase (PARP) (CST, #9542; species reactivity to: Human, Mouse, Rat, Monkey), pyruvate kinase (Thermo Fisher Scientific, #PA5‐13789¸ species reactivity to: Human and Mouse), p‐Foxo1 (S256) (CST, #9461); FoxO1 (CST, #9454¸ species reactivity to: Human, Mouse, Rat, Monkey), p‐Akt (S473) (CST, #4060, species reactivity to: Human, Mouse, Rat, Monkey); Akt (CST, #9272, species reactivity to: Human, Mouse, Rat, Monkey)¸ p‐S6 (S240/S244) (CST, #2215¸ species reactivity to: Human, Mouse, Rat, Monkey), S6 (CST, #2217 species reactivity to: Human, Mouse, Rat, Monkey), TSC22 (R&D Systems, #AF3779, species reactivity to mouse and rat), and valosin‐containing protein (VCP) (Abcam, ab11433¸ Recombinant Fragment Protein within Human VCP aa 250–550 is used as an immunogen) diluted in 3% BSA (Thermo Fisher Scientific). After the overnight incubation, membranes were washed three times with TBS‐T for 10 min each to remove unbound primary antibodies. They were then incubated for 1 h at RT with horseradish peroxidase (HRP)‐conjugated secondary antibodies. Following secondary antibody incubation, the membranes were washed again to remove excess secondary antibodies. The immunoblots were developed using ECL western blotting substrate (Sigma #GERPN2209) to detect the chemiluminescent signals. These signals were then visualized and analyzed using the ChemiDoc Imaging System (Bio‐Rad) and quantification of the western blot signal intensities was performed using Image Lab software (Bio‐Rad).

### Subcellular fractionation assay

INS‐1E cells were seeded on 6 cm dish plates. After 24 h, the cells were transfected with 5 μg pcDNA3‐Flag or Flag‐TSC22D1 plasmids using Lipofectamine™ 2000 Transfection Reagent (Invitrogen™, #11668019) and OPTİMEM (Gibco, #31985047). Forty‐eight hours post‐transfection, a subcellular fractionation assay was performed using the NE‐PER Nuclear and Cytoplasmic Extraction Reagents (Thermo Fisher Scientific, #78835) kit. Protein concentration was measured with the BCA Protein Assay Kit by following the manufacturer's instructions (Thermo Fisher Scientific, #23225). The nuclear and cytoplasmic extractions were subsequently analyzed by western blotting.

### Luciferase assay

INS‐1E cells were seeded on 96‐well plates (40.000 cells/well) and were transfected the next day with 50 nm siRNA scramble control (QIAGEN, #1027281), siTSC22D1 (Thermo Fisher Scientific, #RSS363732), and siFoxO1 (Thermo Fisher Scientific, #RSS331473) using Lipofectamine RNAiMAX (Invitrogen, #13778150) and OPTIMEM (Gibco, #31985047) according to the manufacturer's instructions. Twenty‐four hours post‐transfection, medium was replaced with fresh medium and cells were re‐transfected with 50 ng of pcDNA3 or pGL410_Ins2 (kind gift from Kevin Ferreri; Addgene #49058, species mouse) [62] together with Renilla luciferase pRL‐TK (Promega; Madison, WI, USA; # E2241) plasmids using Lipofectamine™ 2000 Transfection Reagent (Invitrogen, #11668019) and OPTIMEM (Gibco, #31985047). Cells were incubated for a further 48 h, and then, luciferase assay was carried out using Dual‐Luciferase® Reporter Assay System (Promega, #E1980) following the manufacturer's instructions. Firefly Luci and Renilla Luci activity were carried out with FLU0star Omega microplate reader.

### RNA sequencing

Steady state: INS‐1E cells were seeded to 12‐well plates (300.000 cell/well), and after 24 h, cells were transfected with 50 nm siRNA scramble control (QIAGEN, #1027281) and TSC22D1 (Thermo Fisher Scientific, #RSS363732) using Lipofectamine RNAiMAX (Invitrogen, #13778150) and OPTIMEM (Gibco, #31985047). 72 h post‐transfection, total RNA was isolated from INS‐1E cells using the QIAzol and RNeasy kit (QIAGEN, #74106) following the manufacturer's instructions. RNA samples were shipped to Novogene for RNA‐Seq analysis.

−/+ glucose stimulation: INS‐1E cells were seeded to 12‐well plates (300.000 cell/well), and after 24 h, cells were transfected with 50 nm control siRNA [scramble control (QIAGEN, #1027281) and TSC22D1 (Thermo Fisher Scientific, #RSS363732)] using Lipofectamine RNAiMAX (Invitrogen, #13778150) and OPTIMEM (Gibco, #31985047). Seventy‐two hours post‐transfection, cells were starved in KRB buffer containing 0.1% BSA for 4 h, followed by a stimulation with or without [20 mm] glucose for 4 h. Total RNA was isolated from INS‐1E cells using the QIAzol and RNeasy kit (QIAGEN, #74106) following the manufacturer's instructions. RNA samples were shipped to Novogene for RNA‐Seq analysis.

#### Bioinformatic analysis of RNA‐sequencing data

Bioinformatic analysis is performed as described in [63]. The adapter sequence from the raw files was removed by using cutadapt 4.1. Raw counts were then aligned to the rat reference genome (mRatBN7.2) using star 2.7.10a. Any genes that have no transcript detected in any samples were removed. Data normalization and differential expression analysis were performed using the DESeq2 (v3.14) R‐Bioconductor. A statistical threshold was set to the adjusted *P*‐value (Padj) < 0.05, and the effect size was set to a log_2_ fold change (FC) of <−0.5 or >0.5. For the KEGG pathway and GO analysis, the enrichKEGG and enrichGO function of R clusterProfiler package was used, respectively.

The interaction network was constructed by embossing statistically significant abundant candidates from Mass Spec along with FoxO1 onto STRING interaction dB for Rattus norvegicus in the Cytoscape platform.

### Proteomics

#### Sample preparation

INS‐1E cells seeded on 10 cm dishes were transiently transfected with pcDNA3‐Flag vector control [10 μg] or Flag‐TSC22D1 [10 μg] plasmids. Forty‐eight hours post‐transfection, cells were prepared for subsequent treatments by washing twice with 1 mL per well of Tanaka Robertson KRB. Postwash, cells were starved in Tanaka Robertson KRB with 0.1% BSA for 1 h at 37 °C. After the starvation period, cells were treated with Tanaka Robertson KRB containing 0.1% BSA and either 2 mm or 20 mm glucose for 1 h at 37 °C, followed by cell lysis and Flag‐IP. The IP eluates were further subjected to tryptic digest applying a modified filter aided sample preparation (FASP) procedure [64, 65]. After protein reduction and alkylation using DTT and iodoacetamide, samples were denatured in UA buffer (8 M urea in 0.1 M Tris/HCl pH 8.5) and centrifuged on a 30 kDa cutoff filter device (Sartorius) and washed thrice with UA buffer and twice with 50 mm ammonium bicarbonate (ABC) as described in [66]. Proteins were proteolyzed for 2 h at room temperature using 0.5 μg Lys‐C (Wako) and subsequently for 16 h at 37 °C using 1 μg trypsin (Promega). Peptides were collected by centrifugation and acidified with 0.5% trifluoroacetic acid (TFA) and used for liquid chromatography‐tandem mass spectrometry (LC‐MS/MS).

#### LC‐MS/MS analysis

Liquid chromatography with tandem mass spectrometry (LC‐MSMS) analysis was performed in data‐dependent acquisition (DDA) mode, as previously described in [67]. MS data were acquired on a Q‐Exactive HF‐X mass spectrometer (Thermo Fisher Scientific) each online coupled to a nano‐RSLC (Ultimate 3000 RSLC; Dionex). Tryptic peptides were automatically loaded on a C18 trap column (300 μm inner diameter (ID) × 5 mm, Acclaim PepMap100 C18, 5 μm, 100 Å, LC Packings) at a flow rate of 30 μL/min. For chromatography, a C18 reversed‐phase analytical column (nanoEase MZ HSS T3 Column, 100 Å, 1.8 μm, 75 μm × 250 mm, Waters) was used at a flow rate of 250 nL·min^−1^ and following a 95‐min nonlinear acetonitrile gradient from 3 to 40% in 0.1% formic acid. The high‐resolution (60 000 full width at half‐maximum) MS spectrum was acquired with a mass range from 300 to 1500 m/z with an automatic gain control target set to 3 × 106 and a maximum of 30 ms injection time. From the MS prescan, the 15 most abundant peptide ions were selected for fragmentation (MS/MS) if at least doubly charged, with a dynamic exclusion of 30 s. MS/MS spectra were recorded at 15000 resolutions with an automatic gain control target set to 5 × 102 and a maximum of 50 ms injection time. The normalized collision energy was 28, and the spectra were recorded in profile mode.

#### Data processing and protein identification

Data processing was performed as described previously in [68]. Proteome Discoverer 2.5 software (Thermo Fisher Scientific, version 2.5.0.400) was used for peptide and protein identification via a database search (Sequest HT search engine) against Swissprot rat database (Release 2020_02, 8096 sequences). Search settings were 10 ppm precursor tolerance, 0.02 Da fragment tolerance, one missed cleavage allowed. Carbamidomethylation of Cys was set as a static modification. Dynamic modifications included deamidation of Asn, Gln, and Arg, oxidation of Pro and Met; and a combination of Met loss with acetylation on protein N terminus. Percolator was used for validating peptide spectrum matches and peptides, accepting only the top‐scoring hit for each spectrum, and satisfying the cutoff values for false discovery rate (FDR) < 1% and posterior error probability <0.01.

The quantification of proteins was based on abundance values for unique peptides. Abundance values were normalized on total peptide amount, and protein abundances were calculated using the Top 3 most abundant peptides. The final protein ratio was calculated using median abundance values. To address missing values, a low‐abundance imputation was performed, and the statistical significance of the ratio change was determined using ANOVA. The mass spectrometry proteomics data have been deposited to the ProteomeXchange Consortium via the PRIDE [69] partner repository with the dataset identifier PXD054957.

### Software and data analysis

qPCR, GSIS, and luciferase assay data were analyzed using GraphPad Prism for indicated statistical analysis in corresponding figure legends. Graphical Abstract and Fig. 8 were created with BioRender.com.

## Conflict of interest

The authors state that they have no conflicts of interest to disclose.

## Author contributions

BE conceived and supervised the project. BE and S. Yıldırım designed and performed the experiments and did data analysis. AM performed data analysis for both RNA‐Seq experiments as well as for mass spectrometry. ACK and S. Hauck performed the mass spectrometry experiments and data analysis. SL, EE, S. Yilmaz, and AW provided experimental support. FK, JS, and S. Herzig contributed to conceptual discussions. BE and S. Yıldırım wrote the manuscript.

## Supporting information


**Table S1.** List of transcripts differentially regulated upon TSC22D1 knockdown in INS‐1E cells under steady state conditions.

**Table S2.** List of transcripts differentially regulated in glucose‐stimulated control – but not in TSC22D1 knockdown cells.

**Table S3.** List of transcripts differentially regulated in glucose‐stimulated TSC22D1 – but not in control knockdown cells.

## Data Availability

The data that support the findings of this study are available from the corresponding author [bilgen.ekim@med.uni-heidelberg.de] upon reasonable request. The mass spectrometry proteomics data have been deposited to the ProteomeXchange Consortium via the PRIDE partner repository with the dataset identifier PXD054957.
